# Variants and Modifications of the Retroauricular Approach Using in Temporomandibular Joint Surgery: A Systematic Review

**DOI:** 10.3390/jcm10102049

**Published:** 2021-05-11

**Authors:** Maciej Sikora, Maciej Chęciński, Zuzanna Nowak, Dariusz Chlubek

**Affiliations:** 1 Department of Maxillofacial Surgery, Hospital of the Ministry of Interior, Wojska Polskiego 51, 25-375 Kielce, Poland; sikora-maciej@wp.pl; 2 Department of Biochemistry and Medical Chemistry, Pomeranian Medical University, Powstańców Wielkopolskich 72, 70-111 Szczecin, Poland; 3 Preventive Medicine Center, Komorowskiego 12, 30-106 Kraków, Poland; maciej@checinscy.pl; 4 StomaDent Non-Public Healthcare Institution, Dental Clinic, Kościuszki 32, 46-320 Praszka, Poland; zuzannaewanowak33@gmail.com

**Keywords:** TMJ surgery, TMJ disorders, TMJ ankylosis, mandibular condyle, retroauricular approach

## Abstract

Introduction: The retroauricular approach (RA) has been developed in order to expose the temporomandibular joint in a way that minimizes the risk of injury to the facial nerve and masks the postoperative scar. One of its characteristics is an excellent posterolateral view of the mandibular head, which allows for the preservation of the lateral temporomandibular joint ligaments in the course of open intracapsular surgery. Aim: The aim of this study is to systematically review the currently used variants and modifications of RA. Materials and Methods: The construction of the following study is based on PICOS and PRISMA protocols. A systematic literature search was performed based on the PubMed and BASE search engines; furthermore the authors performed a more detailed search in the Google Scholar article database as well as a loop search within the references of papers included in the systematic review. Results: Searching medical articles databases, Google Scholar, and references yielded a total of 85 records. First the titles and abstracts were blindly screened which was followed by a full-text eligibility check resulting in eventually including and qualifying 7 articles for detailed analysis. Discussion: All known variants and modifications of RA are characterized by high safety for the facial nerve and an aesthetically hidden scar. There were no reports of auricle necrosis in the collected material. Conclusions: In this systematic review, 2 variants and 2 modifications of RA that allow for open temporomandibular joint surgery have been identified; all of them together cover a large spectrum of indications for joint surgery, including reposition and osteosynthesis of mandibular head fractures, eminoplasty, or eminectomy and treatment of some forms of ankylosis.

## 1. Introduction

The retroauricular approach (RA) is a surgical technique that involves cutting the skin backwards from the auricle. Among numerous indications for making an incision behind the ear, concerning the pathologies of the auricle itself and the retroauricular area, it is possible to use RA for the operation of structures located anteriorly from the auricle [1,2,3,4,5,6,7]. In the course of RA it is possible to reach the mandibular condyle, the zygomatic arch and a very complex temporomandibular joint [5,6,7,8,9]. This joint, apart from a complex system of ligaments, is also surrounded by very delicate branches of the facial nerve, which makes it difficult to safely access anteriorly from the auricle [5,10,11,12].

In the title of the first known description of the RA, Bockenheimer indicates that the aim of developing this approach was to expose the temporomandibular joint in a way that minimizes the risk of injury to the facial nerve and masks the postoperative scar [13]. The above-mentioned still-valid assumptions are expressed in the results of research works emphasizing these and other advantages of RA [4,5,6,7]. Furthermore, the RA technique is characterized by an adequate posterolateral view of the mandibular head, which allows for the preservation of the lateral temporomandibular joint ligaments in the course of an open intracapsular surgery [4,8,9,10].

The original RA is primarily exerted in the open reposition and internal fixation of mandibular head and neck fractures [8,9,10,11]. However, indications for surgery within the temporomandibular joint are more numerous [5,14,15]. The classic course of RA enables an effective treatment in certain cases of the temporomandibular joint ankylosis [5]. Consecutively, among the indications for the use of modified versions of RA, apart from fractures and ankylosis, there are pathologies that require arthroplasty, eminoplasty, and joint disc surgery [14].

## 2. Aim

The aim of this study is to systematically review the currently used variants and modifications of RA. In order to achieve this objective, the retroauricular preparation techniques were compared, and an attempt was made to systematize indications for RA in each presently implemented form.

## 3. Materials and Methods

### 3.1. Eligibility Criteria

Eligibility for this systematic review was developed by using the standard PICOS framework [16]. The specific inclusion and exclusion criteria were adopted based on the 5 extensions of the PICOS acronym, i.e., Population, Intervention, Comparison, Outcomes and Study design [16]. (1) Population: The diagnoses concerning the structures of the joint itself, i.e., articular surfaces, disc, or capsule, were qualified. It has been decided that all studies concerning the pathologies of the temporomandibular joint in animals need to be excluded but the in vitro studies that used phantoms were not disqualified. (2) Intervention: All works in which the authors describe and discuss their approaches as located posteriorly to the auricle were accepted for further analysis. The rejection criterion in the course of further evaluation was the inability to reach any of the above-mentioned structures of the temporomandibular joint using the described approach. Any type of visualization and surgery within the temporomandibular joint was allowed, i.e., open surgery, endoscopic surgery, and robotic surgery. (3) Comparison: A description of any comparative methods was not required. (4) Outcome: The success of therapy was adopted as the sole criterion for the given surgical approach’s effectiveness. Quite divergent results of the treatment procedures were expected due to admitting manuscripts with varying diagnoses to the review. (5) Study design: All kinds of research work were accepted including case studies and in vitro phantom studies. The language criterion was used, i.e., only English-language works were included in the review. Due to over 100 years of RA history, a decision was made to review only the articles published in 2010 or later. The eligibility criteria are summarized in Table 1.

### 3.2. Search Strategy

A systematic literature search was performed on 25 February 2021 based on the PubMed and BASE [17,18] search engines. The MEDLINE and PubMed Central (PMC) medical databases were searched using PubMed, with over 32 million records in total [17]. BASE, i.e., Bielefeld Academic Search Engine, was used to search almost 9000 medical databases, including the journal archives, i.e., a total of about 266 million records [18]. Search strategies were developed in line with the aforementioned eligibility criteria. A very broad framework has been adopted for diagnosis (Population) and procedures (Intervention), including many possible terms related to RA. When referring to the area behind the auricle, both prefixes appearing in the literature, i.e., retro- and post-, were used. The contents of the created search strategies do not include the presence of comparative methods (Comparison), the intervention results (Outcome) or the type of paper (Study design). Due to possible substantial nomenclature discrepancies in the three issues mentioned, it was decided to manually evaluate the articles against those criteria, i.e., Comparison, Outcome, Study design at further stages of qualification. Additionally, an attempt was made to search for not elsewhere indexed papers using the Google engine implemented in Google Scholar. A very similar search strategy as in the case of medical databases was used. Due to the specificity of the standard operation of the Google engine, i.e., returning a huge number of inaccurate results, a restriction in the form of searching the record titles was introduced. Finally, two of the authors (M.C. and Z.N.) made a loop search in the references of papers previously included in the systematic review until the search ended up without any new results. The content of each search strategy is presented in Table 2.

### 3.3. Exclusion Protocol

The analysis of articles qualifying for rejection at the subsequent stages of the systematic review was performed in accordance with the standard PRISMA protocol [19]. In the first stage, the identified records were synthesized using the Rayyan QCRI application [20]. Along these lines, the search results obtained using the PubMed, BASE, and Google engines were combined. Then, the duplicates were removed, and the records were analyzed. The titles and abstracts were blindly screened by two authors of this systematic review (M.C. and Z.N.). Both authors were obliged to provide reasoning for articles’ rejection according to the PICOS criteria. Cohen’s Kappa coefficient was used to determine the assessments’ convergence. In case of arising disputes, i.e., disagreements whether to approve or reject an article, it was processed to the next stage, i.e., full-text evaluation. The same authors performed a blind full-text evaluation, obtaining the full results convergence. In the next stage, a loop reference search was carried out (M.C. and Z.N.). The final qualification of the papers took place after repeating the PRISMA protocol several times as a result of gradual inclusion and rejection of subsequent records found in the references.

### 3.4. Data Extraction

The stage of obtaining data from the selected articles’ content was carried out in accordance with the general principles of systematic reviews and the objectives of this study. Initially, the two authors qualified articles based on their constitution (M.C. and Z.N.). Then, the three authors pursued an independent data research of the qualified articles’ content characterizing the individual variants and modifications of RA (M.S., M.C. and Z.N.). Afterwards, the results were discussed, appointed, and tabulated. In the next stage, an attempt was made to name and present the previously observed variants and modifications of the discussed surgical access, as well as to indicate the existing differences between them (M.S., M.C. and Z.N.). Finally, based on the literature, the known indications for the use of individual RA variations (M.S., M.C. and Z.N.) were listed. Due to the lack of numerical data and a small number of papers repeatedly referring to a single variant or modification of RA, no meta-analysis of the collected data was undertaken.

## 4. Results

### 4.1. Study Selection

**Figure 1:**
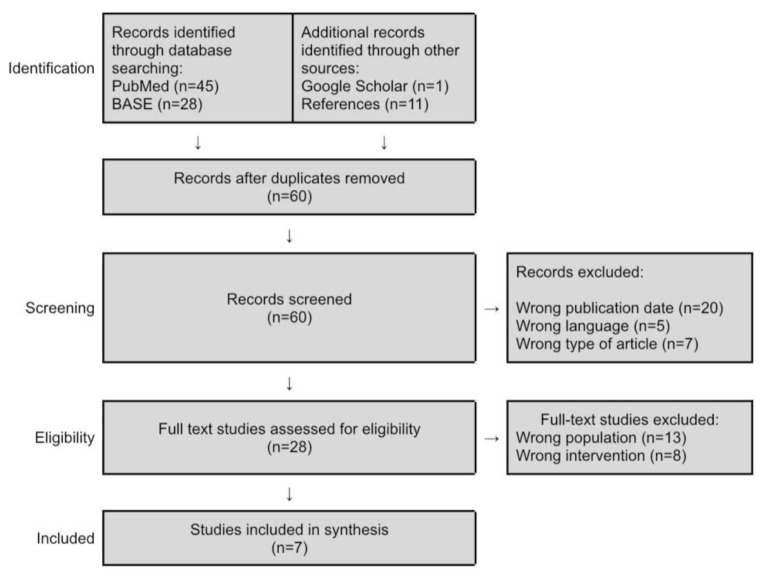
PRISMA flowchart [19]. Description in the text.

Searching medical databases, Google Scholar article databases, and references yielded a total of 85 records. The subsequent selection steps according to the PRISMA protocol are presented in Figure 1 [19].

The use of the diagram and the content of the footnote were made in accordance with the copyright law and do not require changes:

“The PRISMA Statement and the PRISMA Explanation and Elaboration document are distributed under the terms of the Creative Commons Attribution License, which permits unrestricted use, distribution, and reproduction in any medium, provided the original author and source are credited.”

Source: http://prisma-statement.org/prismastatement/CitingAndUsingPRISMA.aspx (accessed on 11 May 2021)

Original author and source is [19]. Source: http://prisma-statement.org/prismastatement/PRISMAStatement.aspx (accessed on 11 May 2021).

### 4.2. Included Studies

The articles included and qualified for detailed analysis are listed in Table 3 in the reverse chronological order.

### 4.3. Quality Assessment

The above-mentioned studies were qualitatively assessed, regarding the study design, the number of patients treated with RA, the presence and type of a control group, and finally the level of evidence. The results of this analysis are presented in Table 4.

### 4.4. Risk of Bias

Randomized case control studies are missing from this systematic review. Thus, the highest level of evidence is in the cohort studies by Kolk et al. and Bansal et al. [4,5]. All the collected material concerns papers with a high risk of bias, including case control studies and even case reports [6,7,15,21]. Therefore, the results presented below are intended for experienced surgeons and should only be taken as suggestions and not expressions of evidence-based medicine.

### 4.5. Comparison

The details of the RA variation used in each study were assessed. The course of cutaneous incision, preparation method, procedures performed on the temporomandibular joint and key stages of wound closure were analyzed. The results of the comparison of the individual RA descriptions are presented in Table 5.

## 5. Discussion

It is assumed that the first description of RA was published by Bockenheimer in 1920, and in the form modified and presented in 1931 by Axhausen, this approach is used to the present day [4,11,13]. The subsequent mentions of RA found in the course of this review were: Aubry (1945), Chiossone (1965), Hoopes (1970), Alexander (1975), Eggleston (1978), Kreutziger (1984), Bramley (1990), Reich (1990), and Donlon (1992). Based on the tabular characteristics of RA variants and modifications presented in the Results section, an attempt was made to synthesize contemporary data regarding the RA.

The original descriptions of RA by Bockenheimer and Axhausen are presented by Kolk, Neff et al. as the source of the surgical technique used in the analyzed study [4,13]. It is known that the RA was widely popularized by Neff, the second author of the work of Kolk et al. and the first author of numerous studies on the fixation of the mandibular head using RA [8,11]. Bearing in mind that the two operators in the paper by Kolk et al. were Neff and Kolk, it should be assumed that the lack of detailed surgical technique description in the discussed text may result from its earlier presentation in other publications. Therefore, for the purposes of this systematic review, it was decided that the original RA technique should be distinguished and some of its aspects based on Neff’s description in the English-language academic textbooks have to be noted [8,9]. Further analysis of the included studies showed that Bansal et al. used the same version of RA [5].

During the preparation of previous studies on RA and in the course of the previous stages of this review, it was found that the articles by Benech et al. and Arcuri et al. clearly refer to the same study group, and the descriptions of the surgical technique in both of them seem to be consistent [6,7,10]. Therefore, a decision to combine these two descriptions in a complementary way was made and to refer to them as the Italian variant of RA due to the affiliation of the authors.

An extended access described by Blythe is termed “the temporal RA extension” in the further contents of this discussion [15]. In the case of the bat wing approach, the original nomenclature was retained [14]. In the course of an extensive ablation surgery described by Yokoyama et al., RA was only a fragment of the approach, and the temporomandibular joint constituted only a part of the resected tissues, so this case will not be analyzed any further [21]. In regard to the above comments, the data collected from the different RA applications was combined into 2 main variants and 2 modifications, as shown in Table 6.

### 5.1. RA Variants

#### 5.1.1. Original RA Technique

We owe numerous descriptions of RA to Neff, who repeatedly emphasized that it was based on the original German-language works by Bockenheimer and Axhausen [4,8,9]. There is no doubt that the approach has undergone numerous improvements since 1920, but it must be admitted that it was Neff who was the main promoter of RA in Germany in recent years [11]. Therefore, the English descriptions of the RA technique presented by Neff were considered as reference for the purposes of this study [8,9,11].

The incision is made along almost the entire auricle length, 2–3 mm backwards from the postauricular sulcus [8,9]. The preparation is performed on the mastoid and the deep temporal fascias [8,9]. The external auditory meatus is transected with a single cut and the medial temporal vessels are ligated and transected [9]. Any injury to the lateral ligament of the temporomandibular joint and the lateral part of the joint capsule should be avoided [8,9]. The obtained insight allows for the fixation of the fractures of the mandibular head, preferably with the use of a small fragment positional screws [4,8,9]. To avoid the external auditory canal stenosis, the auricle is positioned posteriorly using basal sutures [8]. The external auditory canal is reconstructed using prelaid sutures [8]. The above assumption does not exhaust the complex subject of the RA technique, descriptions of which Neff drew in part from Reich, elaborated on, and published in detail [8,9,23]. This discussion focuses only on some key stages, the highlighting of which is to further compare the technique with that used by other authors.

The study by Kolk, Neff et al. described a group of 62 patients, 22 of whom were closely followed over the long term; based on this study, general conclusions were drawn about the results of the classical version of RA [4]. Temporary hypofunction of the temporal branch of the facial nerve is rare and amounts to approximately 3.5% of cases [4]. There are no reports of permanent damage to the facial nerve [4]. Transient hypoaesthesia within auriculotemporal and great auricular innervation areas occur in over 40% of patients [4]. Persistent auriculotemporal hypoaesthesia occurs in 1 in 10 patients on average [4]. Stenosis of the external auditory canal is found in about 1 in 3 patients, but it is no greater than 1.5 mm of reduction in diameter [4]. The typical external auditory canal diameter in an adult is about 8 mm, which means the narrowing does not exceed 20% in the classic version of RA [4,24].

The article by Bansal et al. describes the effective treatment of ankylosis in 15 temporomandibular joints via RA [5]. Hypofunction of the temporal branch of the facial nerve was present in approximately 13% the joints operated via RA, and it was transient in all cases [5]. Moreover, in 60% of RA cases transient stenosis of the external auditory canal has been reported [5]. Thus, slight deterioration of hearing occurred in about half of the cases and each time it resolved after up to 2 months post-surgery [5].

#### 5.1.2. Italian RA Variant

Benech, Arcuri et al., when introducing the RA to the array of operations performed, were relying on a German-language article by Neff et al. from 2004 as well as some earlier sources [6,7,25]. According to the authors of the Italian variant, they had hardly any modern English-language descriptions of the operational technique of RA at their disposal [6,7]. This is probably what led to the development of the Italian variant of RA in parallel with the acquisition of further experiences by Neff, Kolk et al.

The skin incision is made on the auricle, about 10–15 mm from the postauricular sulcus, so it can be even 10 mm shorter from the top and up to 10 mm shorter from the auricular lobe than the sulcus itself [6,7]. The preparation is performed on the auricle perichondrium and on the mastoid fascia [6,7]. The external auditory canal is transected in two steps, the retromandibular vein is always isolated, ligated, and transected [6,7]. The osteosynthesis is performed using miniplates and screws [6]. Three basal sutures are placed to position the auricle and avoid the external auditory canal stenosis [7]. Instead of prelaid sutures, absorbable sutures are used [6,7].

Benech, Arcuri et al. found no case of permanent dysfunction of the facial and auriculotemporal nerves in a group of 14 patients [6,7]. However, a single case of temporary hypofunction of the facial nerve frontal branch was reported [6,7]. There were no cases of the external auditory meatus stenosis [6,7].

### 5.2. RA Modifications

The essential shape of the incision in RA runs almost the full length of the sulcus of the retroauricular crease [8,11]. According to the publications which underwent full-text analysis in the course of this review, the incision can be extended in three directions: (a) upper-anterior; (b) lower-anterior; and (c) posterior [14,26,27]. Out of these, only the first, upper-anterior extension is used for a wider exposure of the temporomandibular joint [14,15]. The next two, i.e., the lower-anterior and posterior modifications of RA, assume the shortening of the main incision by its upper part corresponding to approximately half the length of the original preparation [26,27]. The shortening of the upper part of the incision, the limitation of the subauricular preparation, and the lack of cutting the external auditory canal result in the inability to reach the temporomandibular joint.

#### 5.2.1. Lower RA Modifications

The anterior extension of the lower part of the RA with the shortening of its upper part leads to the “minimal incision approach”, which is also a retroauricular modification of the perilobular approach [27]. The “minimal incision approach” is used for parotidectomy or reposition with internal fixation of the mandibular neck fractures [27]. The posterior modification of RA, referred to simply as the “retroauricular approach”, is primarily a modification of the rhytidectomy approach in the form of shortening the original incision by the preaural fragment [26,28]. As a result of the limitation of the cutaneous incision, the open view into the surgical field deteriorates in relation to the original rhytidectomy approach [26,28]. The posterior RA modification is used for endoscopic and robotic neck dissections, the reconstruction of post-oncological mandibular defects with free flaps attached to the jugular vessels and to perform excision of the parotid or submandibular glands [26,29,30,31].

In the course of this review, descriptions of downward extension of RA while retaining the full length of the original incision were not noted. When analyzing the blood supply to the auricule, it can be assumed that such modifications could lead to ischemia or even auricle necrosis [32]. The auricle is supplied with blood from the posterior auricular artery and the three anterior auricular arteries departing from the superficial temporal artery [32]. All described arteries are vulnerable to injury in commonly used approaches, e.g., rhytidectomy approach [26,28,32]. Nevertheless, in the rhytidectomy approach an extensive cutaneous peduncle vascularizing the auricle from the top and back is preserved, and there is no preparation under the auricle nor cutting the external auditory meatus [26,28].

#### 5.2.2. Temporal RA Extension

Two upper modifications of RA are known, both of which are extending the cut temporally and anteriorly [14,15]. First modification, presented by Blythe et al. and proposed by Kreutziger in 1987, differs from the classical course of RA mainly due to the extension of the incision [15,22]. The preparation still includes transection of the external auditory canal and the absence or presence of sutures retracting the auricle seems to be an individual decision of an operator [15]. It has been proven that a more extensive open view of the operating field allows for the removal of fibro-osseous adhesions as well as even a simple reconstruction aimed at restoring the function of the joint [15]. It may be assumed that temporally extended RA also allows for other manipulations within the temporomandibular joint, which, however, was not discussed by Blythe et al. [15]. Albeit, the original description of the approach, then referred to by Kreutziger as extended modified postauricular incision, presents a case of the temporomandibular joint plasty, including condylectomy and eminoplasty [22]. Due to isolated cases described by Kreutziger and Blythe et al., it is difficult to assess the safety of the temporal extension of RA [15,22]. Nevertheless, in both studies authors emphasize the high safety of the facial nerve branches [15,22].

#### 5.2.3. Bat Wing Approach

A far-reaching modification of RA was one described by Garcia y Sanchez et al., with a separate name of the “bat wing approach” [14]. This access was proposed by the same author back in 1993 and was used since then [14]. In contrast to the variants and modifications discussed above, this time the external ear canal is spared [14]. The lack of transection of the external auditory meatus does not prevent reaching the temporomandibular joint, which, according to the author’s assurances, enables not only numerous plasty of the joint structures, including even eminectomy, but also the fixation of fractures of the upper part of the mandibular head [14].

Garcia y Sanchez et al. experienced no damage to the palpebral branch of the facial nerve in the course of observation in the years 1993–2015, due to its topographic location [14,33]. Exact measurements relative to bony structures were therefore described by these authors [14]. Nevertheless, the palpebral branch is the most vulnerable of all facial nerve branches in course of the bat wing approach [14,33,34]. The auriculotemporal nerve is another essential structure located in this area [35]. Therefore, a high risk of injury to the auriculotemporal nerve when using the bat wing approach results from the anatomy of the nerve and the approach’s design [14,35]. However, in the described observation period Garcia y Sanchez did not mention any hypoesthesia related to auriculotemporal nerve hypofunction [14].

### 5.3. Indications and Contraindications

Among the indications for the use of RA, there are no diagnostic surgeries. The assessment of temporomandibular joint dysfunctions should be performed based on the clinical examination and additional imagining, such as magnetic resonance imaging [36,37]. Furthermore, arthroscopy may be used as a supplement to these methods [36,38]. Therefore, the indications for RA are purely therapeutic.

The current articles and academic textbooks are presenting the original RA as one of the possible methods of open surgery for a fractured uppermost portion of the mandibular condyle, i.e., the mandibular head and the upper part of the mandibular neck [10,11]. Lower mandibular condyle fractures cannot be visualized well with even a modified RA and require the use of other approaches [14,39]. Detailed classifications of the mandibular condyles’ anatomy for surgical purposes helps in assessing the possibility of using the RA for fixation and choosing the fixation method [40,41]. The use of unmodified RA in cases of mandibular condyle fractures was discussed in detail in one of our previous papers (Sikora et al. 2021) [10]. Arthroplasty and ankylosis surgery and other possible indications for the use of RA and its variants and modifications [5,14,15] and the confirmed indications and contraindications for individual types of RA discussed are presented together in Table 7.

### 5.4. Safety of RA

A common feature of all RA management strategies seems to be high safety for the facial nerve [4,6,7,14,15]. No permanent damage to the facial nerve was found in any of the cohort and case control studies [4,5,6,7,14]. Regarding the sensory nerves, only Kolk et al. reported permanent auriculotemporal hypoaesthesia [4]. The lack of this complication in the studies of other authors may, however, not only be related to the surgical technique, but also result from the method of observation, which was the most meticulous in the study by Kolk et al. [4]. None of the screened abstracts nor any of the full-text articles analyzed in this systematic review have shown any complications in the form of transient or permanent auricle ischemia due to the RA. Therefore, there are no known cases of auricle necrosis as a consequence of surgery via RA.

Among the above-described indications for the use of RA, i.e., the treatment of ankylosis, various types of arthroplasty, and fixation of the uppermost part of the mandibular condyle fractions, only the latter indication calls for a double routine performance of the RA [4,5,6,7,10,14,15]. The routine second surgical intervention in the treatment of fractures is to remove the fixation material [4,10]. In order to avoid the reoperation, a research was undertaken on the possibility of using fixation screws made of resorbable materials [42,43]. Promising results from recent studies indicate that the use of magnesium alloy screws can solve the issue of double operations and thus reduce postoperative complications [42,43].

## 6. Conclusions

The original RA technique is primarily used in the mandibular head fracture surgery but can also be applied in the treatment of initial stages of ankylosis. It is very safe for the facial nerve and the scar placement is highly aesthetic, which distinguishes it from the preauricular approaches typically used under these indications. The Italian RA variant, so far employed in the mandibular head surgery, seems to reduce the percentage of postoperative ear canal stenosis, while maintaining the above-mentioned advantages. Two temporally released RA modifications extend the indications for RA to include eminoplasty and eminectomy-sparing or cutting the ear canal is optional for these operations. Our systematic literature review suggests that all variants and modifications of the RA are highly effective in open joint surgery. Medical professionals that specialize in TMJ surgery should be aware of possible RA approaches, along with their variants and modifications, in order to apply the best possible solution for treating their own patients.

## Figures and Tables

**Table 1:** Short version of the eligibility criteria. Full description in the text.

	Inclusion Criteria	Exclusion Criteria
Population	Pathologies of the temporomandibular joint	Animal patients
Intervention	Any variants and modifications of the RA	Approaches not reaching temporomandibular joint for any reason, including other objective or failure
Comparison	Any or none	-
Outcome	Effective treatment of the temporomandibular joint pathology via RA	-
Study design	Research articles and case reports published in English	Papers published prior to 2010

**Table 2:** Applied search strategies. Description in the text.

	Search Strategy
PubMed	(temporomandibular OR mandible OR mandibular) AND (retroauricular OR retro-auricular OR retroaural OR retro-aural OR postauricular OR post-auricular OR postaural OR post-aural OR transmeatal OR trans-meatal) AND (approach OR access)
BASE	(temporomandibular mandible mandibular) AND (retroauricular retro-auricular retroaural retro-aural postauricular post-auricular postaural post-aural transmeatal trans-meatal) AND (approach access)
Google Scholar	allintitle: (temporomandibular mandible OR mandibular) (retroauricular OR retro-auricular OR retroaural OR retro-aural OR postauricular OR post-auricular OR postaural OR post-aural OR transmeatal OR trans-meatal) (approach OR access)

**Table 3:** Studies included as a result of the systematic review.

Publication Date	First Author	Title
2016	Blythe et al. [15]	Extended retroauricular access to the medial temporomandibular joint space
2015	Garcia y Sanchez et al. [14]	Bat wing surgical approach for the temporomandibular joint
2015	Kolk et al. [4]	Long-term results of ORIF of condylar head fractures of the mandible: A prospective 5-year follow-up study of small-fragment positional-screw osteosynthesis (SFPSO)
2012	Bansal et al. [5]	The post-auricular approach for gap arthroplasty—a clinical investigation
2012	Arcuri et al. [7]	Analysis of the retroauricular transmeatal approach: a novel transfacial access to the mandibular skeleton
2011	Benech et al. [6]	Retroauricular transmeatal approach to manage mandibular condylar head fractures
2011	Yokoyama et al. [21]	Successful function-preserving therapy for chondroblastoma of the temporal bone involving the temporomandibular joint

**Table 4:** Quality assessment. Description in the text. * The study involved 15 patients operated bilaterally, i.e., 30 approaches were performed, 15 of which were RA. The exact number of patients who underwent RA was not reported.

First Author and Publication Date	Study Design	Number of Patients	Control Group	Level of Evidence
Blythe et al., 2016 [15]	Case report	1	-	4
Garcia y Sanchez et al., 2015 [14]	Retrospective	20	-	3
Kolk et al., 2015 [4]	Prospective	62 (22 underwent full evaluation)	Retrospective; unspecified approach	2
Bansal et al., 2012 [5]	Prospective	8–15 *	Preauricular approach	2
Arcuri et al., 2012 [7]	Retrospective	14	-	3
Benech et al., 2011 [6]	Retrospective	14	-	3
Yokoyama et al., 2011 [21]	Case report	1	-	4

**Table 5:** Comparison of surgical techniques presented in individual studies.

First Author and Publication Date	Skin Incision	Preparation	Procedures	Wound Closure
Blythe et al., 2016 [15]	Main incision: from the top of the auricle to the earlobe; 2 mm anteriorly to the postauricular sulcus Extension: from the top of the auricule towards the superior temporal line; curvilinear; 80 mm long	Preparation on: temporal fascia, auricular cartilage and mastoid periosteum External auditory meatus transection: yes	Removal of the medially displaced condylar heads and ankylotic masses Reconstruction of the temporomandibular joint with autologous cartilage transplant	Auricle-retracting sutures: no External auditory meatus suturing: absorbable sutures
Garcia y Sanchez et al., 2015 [14]	Main incision: from the top of the auricule towards the earlobe; 5 mm posteriorly to the postauricular sulcus; 25 mm long Extension: from the top of the auricule towards the superior temporal line; curvilinear; 25 mm long	Preparation on: bone External auditory meatus transection: no	Eminoplasty and eminectomy	Auricle-retracting sutures: no External auditory meatus suturing: not applicable
Kolk et al., 2015 [4]	Not specified	Not specified	ORIF of the mandibular head fractures with small fragment positional screws	Not specified
Bansal et al., 2012 [5]	Main incision: from the top of the auricle to the earlobe; 3–4 mm posteriorly to the postauricular sulcus Extension: no	Preparation on: mastoid and temporalis fascia External auditory meatus transection: yes	Gap arthroplasty due to temporomandibular joint ankylosis	Auricle-retracting sutures: yes External auditory meatus suturing: prelaid sutures
Arcuri et al., 2012 [7]	Main incision: 25–30 mm in length; 10–15 mm anteriorly to the postauricular sulcus Extension: no	Preparation on: auricle perichondrium and mastoid fascia External auditory meatus transection: yes	ORIF of the mandibular head fractures with miniplates	Auricle-retracting sutures: yes External auditory meatus suturing: absorbable sutures
Benech et al., 2011 [6]	Main incision: 25–30 mm in length; 10–15 mm anteriorly to the postauricular sulcus Extension: no	Preparation on: auricle perichondrium and mastoid fascia External auditory meatus transection: yes	ORIF of the mandibular head fractures with miniplates	Auricle-retracting sutures: not specified External auditory meatus suturing: absorbable sutures
Yokoyama et al., 2011 [21]	Main incision: from the top of the auricule towards the earlobe; otherwise not specified Extension: from the bottom of the auricle towards the neck	Preparation on: not specified fascias External auditory meatus transection: not specified	Resection of the temporomandibular joint in the course of extensive oncological surgery	Not specified

**Table 6:** Synthesized data on RA variants and modifications. ORIF-open reposition and internal fixation.

Variant or Modification	Source Authors	Incision Extension	Preparation	Procedures	Auricle-Retracting Sutures	External Auditory Meatus Suturing
Original RA technique [4,5,8,9]	Neff Bansal et al.	No extension	Transmeatal	ORIF of the mandibular head fractures with small fragment positional screws Ankylosis treatment	Basal sutures	Prelaid sutures
Italian RA variant [6,7]	Benech et al. Arcuri et al.	No extension	Transmeatal	ORIF of the mandibular head fractures with miniplates	Basal sutures	Absorbable sutures
Temporal RA extension [15,22]	Blythe et al. Kreutziger	Temporally extended	Transmeatal	Ankylosis treatment	None	Absorbable sutures
Bat wing approach [14]	Garcia y Sanchez et al.	Temporally extended	Bypassing the external auditory meatus from above	Eminoplasty and eminectomy	None	Not applicable

**Table 7:** Indications (Yes) and contraindications (No) specified in the content of the included studies. Boxes without data (-) indicate no mention in the articles included in this systematic review.

	Condylar Head Fracture	Eminoplasty or Eminectomy	Temporomandibular Joint Ankylosis
Original RA technique [4,5,8,9]	Yes	-	Yes, ankylosis within the joint capsule
Italian RA variant [6,7]	Yes	-	-
Temporal RA extension [15,22]	-	Yes	Yes, fibro-osseous ankylosis
Bat wing approach [14]	Yes, fractures of the upper third of the condylar head	Yes	No
